# 4′-Acetyl-3′′-carbamoyl-[1,1′:3′,1′′-terphen­yl]-2-carb­oxy­lic acid

**DOI:** 10.1107/S1600536813026482

**Published:** 2013-09-28

**Authors:** Yoshinobu Ishikawa, Nanako Yoshida, Takafumi Suzuki

**Affiliations:** a School of Pharmaceutical Sciences, University of Shizuoka, 52-1 Yada, Suruga-ku, Shizuoka 422-8526, Japan

## Abstract

In the title *m*-terphenyl derivative, C_22_H_17_NO_4_, the dihedral angles between the aromatic rings of the benzoic acid–acetophenone, acetophenone–benzamide and benzoic acid–benzamide units are 45.39 (8), 48.02 (8) and 42.93 (8)°, respectively. The carbamoyl and carboxyl groups are disordered with a refined occupancy ratio of 0.558 (15):0.442 (15). In the crystal, mol­ecules are linked through O—H⋯O and N—H⋯O hydrogen bonds between terminal carboxyl and carbamoyl groups in a bidentate manner, and anti­parallel helices are formed which extend along the *b*-axis direction.

## Related literature

For background to this study, see: Ishikawa & Fujii (2011 ▶). For related structures, see: Schnobrich *et al.* (2010 ▶); Fun *et al.* (2012 ▶); Liu *et al.* (2013 ▶). For the biological activity of a related compound, see: Tomassini *et al.* (1994 ▶).
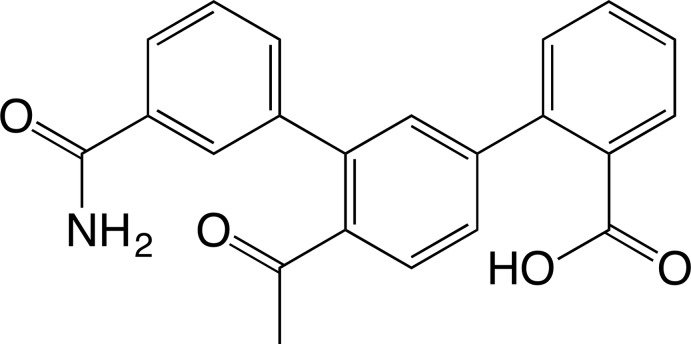


## Experimental

### 

#### Crystal data

C_22_H_17_NO_4_
*M* *_r_* = 359.38
Monoclinic, 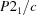

*a* = 13.076 (4) Å
*b* = 20.581 (5) Å
*c* = 6.725 (3) Å
β = 104.10 (3)°
*V* = 1755.2 (9) Å^3^
*Z* = 4
Mo *K*α radiation
μ = 0.09 mm^−1^
*T* = 100 K
0.38 × 0.25 × 0.20 mm

#### Data collection

Rigaku AFC-7R diffractometer
4953 measured reflections
4026 independent reflections
2389 reflections with *F* ^2^ > 2σ(*F* ^2^)
*R*_int_ = 0.014
3 standard reflections every 150 reflections intensity decay: 0.6%

#### Refinement

*R*[*F* ^2^ > 2σ(*F* ^2^)] = 0.050
*wR*(*F* ^2^) = 0.132
*S* = 1.01
4026 reflections
284 parameters
10 restraints
H-atom parameters constrained
Δρ_max_ = 0.23 e Å^−3^
Δρ_min_ = −0.34 e Å^−3^

### 

Data collection: *WinAFC* (Rigaku, 1999 ▶); cell refinement: *WinAFC*; data reduction: *WinAFC*; program(s) used to solve structure: *SIR2008* (Burla *et al.*, 2007 ▶); program(s) used to refine structure: *SHELXL97* (Sheldrick, 2008 ▶); molecular graphics: *CrystalStructure* (Rigaku, 2010 ▶); software used to prepare material for publication: *CrystalStructure*.

## Supplementary Material

Crystal structure: contains datablock(s) General, I. DOI: 10.1107/S1600536813026482/rn2118sup1.cif

Structure factors: contains datablock(s) I. DOI: 10.1107/S1600536813026482/rn2118Isup2.hkl

Supplementary material file. DOI: 10.1107/S1600536813026482/rn2118Isup3.cml

Additional supplementary materials:  crystallographic information; 3D view; checkCIF report

## Figures and Tables

**Table 1:** Hydrogen-bond geometry (Å, °)

*D*—H⋯*A*	*D*—H	H⋯*A*	*D*⋯*A*	*D*—H⋯*A*
O25*A*—H25*A*⋯O27*A* ^i^	0.84	1.72	2.544 (9)	168
O25*B*—H25*B*⋯O27*B* ^i^	0.84	1.72	2.546 (8)	167
N23*A*—H23*A*⋯O24*A* ^ii^	0.88	1.80	2.661 (8)	166
N23*B*—H23*C*⋯O24*B* ^ii^	0.88	1.83	2.667 (10)	161

Symmetry codes: (i) 
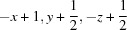
; (ii) 
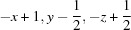
.

**Fig. 1.**
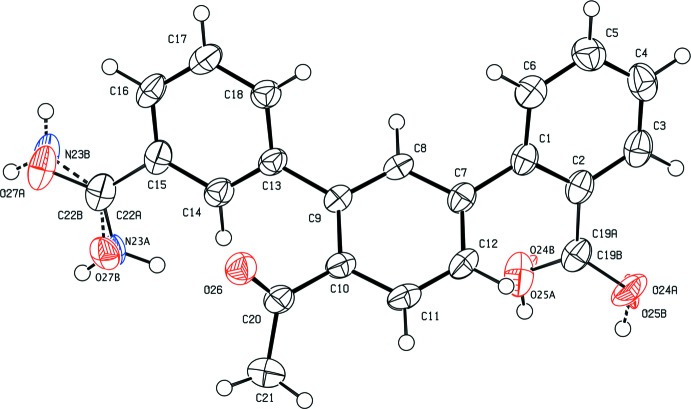
The molecular structure of the title compound, with displacement ellipsoids drawn at the 50% probability level. Hydrogen atoms are shown as small spheres of arbitrary radius. Disordered atoms are discriminated with A/B notation, and the minor disordered form B is drawn as dashed bonds.

**Fig. 2.**
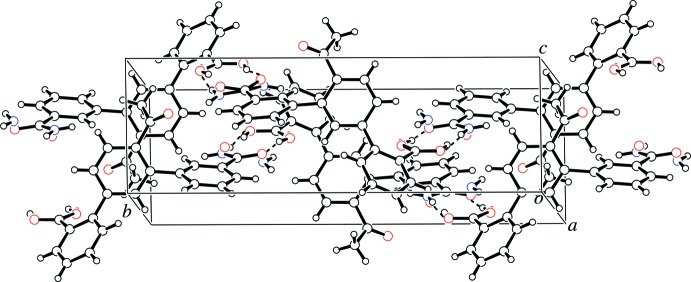
A crystal packing view of the title compound. Intermolecular O–H···O and N–H···O hydrogen bonds are represented by dashed lines only for the disordered form A for clarity.

**Fig. 3.**
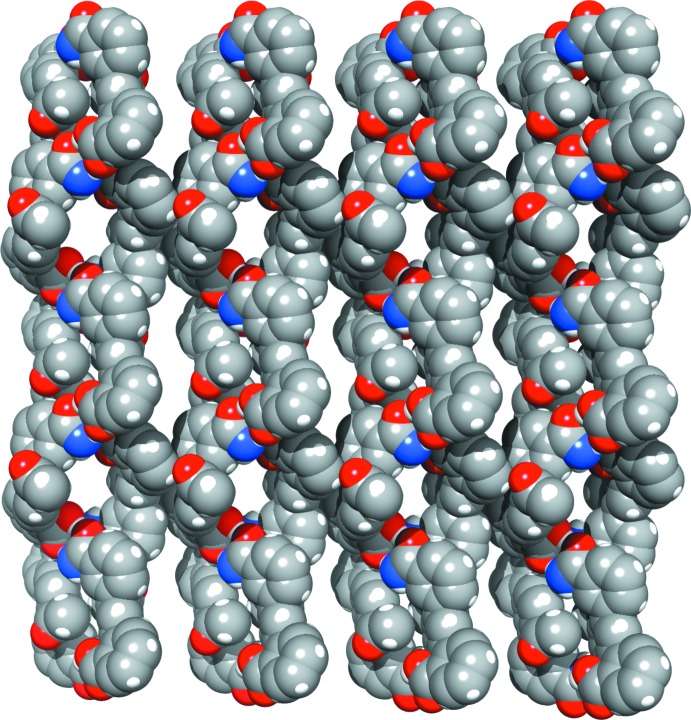
A crystal packing view of the title compound represented by a sphere model. Anti-parallel helices align parallel to the b axis. The disordered form B is not shown for clarity.

## supplementary crystallographic information

### 1. Comment

Aryl diketo acids are known to inhibit influenza virus endonuclease (Tomassini *et al.* 1994). According to our inhibitor design targeting this metalloenzyme (Ishikawa & Fujii 2011), we synthesized the title compound by hydrolysis of methyl 4'-acetyl-3''-carbamoyl-[1,1':3',1''-terphenyl]-2-carboxylate, where the *m*-terphenyl derivatives are the synthetic intermediates of a final *m*-terphenyl diketo acid. The dihedral angles between A (C1/C2/C3/C4/C5/C6) and B (C7/C8/C9/C10/C11/C12), B and C (C13/C14/C15/C16/C17/C18), and A and C rings are 45.39 (8), 48.02 (8) and 42.93 (8)°, respectively (Fig. 1). The carbamoyl and carboxyl groups are disordered with a refined occupancy ratio of 0.558 (15) (A): 0.442 (15) (B). In the crystal, anti-parallel helices are formed through intermolecular O–H···O and N–H···O hydrogen bonds between terminal carboxylic acid and carbamoyl groups in a bidentate manner (Table 1), and align parallel to the *b* axis (Fig. 2 and 3).

### 2. Experimental

Methyl 4'-acetyl-3''-carbamoyl-[1,1':3',1''-terphenyl]-2-carboxylate (2.70 mmol) was dissolved in a mixture of 10 M NaOH (40 ml), 2-propanol (20 ml) and THF (20 ml), which was then refluxed overnight. The reaction mixture was cooled to room temperature, and acidified (pH = 4) with 2 M HCl. After the mixture was extracted with ethyl acetate, the organic layer was washed with water and brine, and dried over anhydrous Na_2_SO_4_. Concentration of the organic solution gave the title compound (yield 94%). Single crystals suitable for X-ray diffraction were obtained by slow evaporation of an acetone/*n*-hexane solution of the compound at room temperature.

### 3. Refinement

The hydrogen atoms of phenyl groups were placed in geometrical positions [C–H 0.95 Å, *U*_iso_(H) = 1.2*U*_eq_(C)], and refined using a riding model. Hydrogen atoms of methyl group were found in a difference Fourier map, and a rotating group model was applied with distance constraint [C–H = 0.98 Å, *U*_iso_(H) = 1.2*U*_eq_(C)]. The carbamoyl and carboxyl groups were disordered, and the occupancies of carbamoyl and carboxyl groups were determined to 0.558 (15) for A and 0.442 (15) for B. Hydroxyl hydrogen atoms for both parts, A and B, were found in a difference Fourier map, and a rotating group model was applied with distance constraint [O–H 0.84 Å, *U*_iso_(H) = 1.2*U*_eq_(O)]. The hydrogen atoms of A and B parts of carbamoyl group were placed in geometrical positions [N–H 0.88 Å, *U*_iso_(H) = 1.2*U*_eq_(N)], and refined using a riding model.

### Crystal data

**Table:**

C_22_H_17_NO_4_	*F*(000) = 752.00
*M**_r_* = 359.38	*D*_x_ = 1.360 Mg m^−^^3^
Monoclinic, *P*2_1_/*c*	Mo *K*α radiation, λ = 0.71069 Å
Hall symbol: -P 2ybc	Cell parameters from 25 reflections
*a* = 13.076 (4) Å	θ = 16.0–17.4°
*b* = 20.581 (5) Å	µ = 0.09 mm^−^^1^
*c* = 6.725 (3) Å	*T* = 100 K
β = 104.10 (3)°	Plate, colorless
*V* = 1755.2 (9) Å^3^	0.38 × 0.25 × 0.20 mm
*Z* = 4	

### Data collection

**Table:**

Rigaku AFC-7R diffractometer	θ_max_ = 27.5°
ω scans	*h* = −16→16
4953 measured reflections	*k* = 0→26
4026 independent reflections	*l* = −8→4
2389 reflections with *F*^2^ > 2σ(*F*^2^)	3 standard reflections every 150 reflections
*R*_int_ = 0.014	intensity decay: 0.6%

### Refinement

**Table:**

Refinement on *F*^2^	Secondary atom site location: difference Fourier map
*R*[*F*^2^ > 2σ(*F*^2^)] = 0.050	Hydrogen site location: inferred from neighbouring sites
*wR*(*F*^2^) = 0.132	H-atom parameters constrained
*S* = 1.01	*w* = 1/[σ^2^(*F*_o_^2^) + (0.0495*P*)^2^ + 0.6018*P*] where *P* = (*F*_o_^2^ + 2*F*_c_^2^)/3
4026 reflections	(Δ/σ)_max_ < 0.001
284 parameters	Δρ_max_ = 0.23 e Å^−^^3^
10 restraints	Δρ_min_ = −0.34 e Å^−^^3^
Primary atom site location: structure-invariant direct methods	

### Special details

**Table:**

Refinement. Refinement was performed using all reflections. The weighted *R*-factor (*wR*) and goodness of fit (*S*) are based on *F*^2^. *R*-factor (gt) are based on *F*. The threshold expression of *F*^2^ > 2.0 σ(*F*^2^) is used only for calculating *R*-factor (gt).

### Fractional atomic coordinates and isotropic or equivalent isotropic displacement parameters (Å^2^)

**Table:**

	*x*	*y*	*z*	*U*_iso_*/*U*_eq_	Occ. (<1)
O24A	0.7288 (6)	0.7406 (4)	0.6288 (14)	0.053 (4)	0.558 (15)
O24B	0.6566 (6)	0.6441 (4)	0.572 (3)	0.040 (4)	0.442 (15)
O25A	0.6540 (7)	0.6429 (4)	0.558 (3)	0.056 (4)	0.558 (15)
O25B	0.7277 (6)	0.7431 (4)	0.6298 (14)	0.029 (3)	0.442 (15)
O26	0.72444 (13)	0.41418 (8)	−0.1531 (3)	0.0419 (4)	
O27A	0.5183 (2)	0.20108 (10)	0.0987 (13)	0.0431 (17)	0.558 (15)
O27B	0.4504 (3)	0.29447 (17)	0.0312 (17)	0.039 (3)	0.442 (15)
N23A	0.44490 (18)	0.30101 (11)	0.0760 (13)	0.0293 (18)	0.558 (15)
N23B	0.5187 (3)	0.19802 (9)	0.1589 (13)	0.036 (2)	0.442 (15)
C1	0.87031 (17)	0.59106 (10)	0.6538 (4)	0.0341 (5)	
C2	0.83737 (17)	0.64963 (10)	0.7265 (4)	0.0365 (6)	
C3	0.89855 (19)	0.67828 (11)	0.9030 (4)	0.0433 (6)	
C4	0.9899 (2)	0.64923 (12)	1.0133 (4)	0.0468 (7)	
C5	1.02260 (19)	0.59176 (12)	0.9444 (4)	0.0465 (6)	
C6	0.96413 (18)	0.56329 (11)	0.7670 (4)	0.0411 (6)	
C7	0.81424 (17)	0.55911 (10)	0.4596 (4)	0.0328 (5)	
C8	0.79962 (16)	0.49204 (10)	0.4560 (4)	0.0317 (5)	
C9	0.75198 (16)	0.45849 (10)	0.2784 (4)	0.0310 (5)	
C10	0.71633 (16)	0.49374 (10)	0.0949 (4)	0.0328 (5)	
C11	0.72897 (18)	0.56122 (10)	0.0999 (4)	0.0390 (6)	
C12	0.77824 (17)	0.59325 (10)	0.2770 (4)	0.0372 (6)	
C13	0.73748 (18)	0.38695 (10)	0.2912 (4)	0.0327 (5)	
C14	0.64108 (18)	0.35803 (10)	0.2106 (4)	0.0347 (5)	
C15	0.62910 (19)	0.29090 (10)	0.2228 (4)	0.0371 (6)	
C16	0.7138 (2)	0.25265 (10)	0.3196 (4)	0.0398 (6)	
C17	0.8098 (2)	0.28157 (11)	0.4053 (4)	0.0412 (6)	
C18	0.82187 (19)	0.34818 (10)	0.3917 (4)	0.0362 (6)	
C19A	0.73405 (18)	0.68032 (8)	0.6318 (4)	0.0383 (6)	0.558 (15)
C19B	0.73405 (18)	0.68032 (8)	0.6318 (4)	0.0383 (6)	0.442 (15)
C20	0.67702 (18)	0.46037 (11)	−0.1066 (4)	0.0375 (6)	
C21	0.5798 (3)	0.48606 (14)	−0.2520 (5)	0.0591 (8)	
C22A	0.52595 (15)	0.26103 (7)	0.1284 (4)	0.0404 (6)	0.558 (15)
C22B	0.52595 (15)	0.26103 (7)	0.1284 (4)	0.0404 (6)	0.442 (15)
H3	0.8771	0.7186	0.9484	0.0520*	
H4	1.0299	0.6687	1.1358	0.0562*	
H5	1.0856	0.5716	1.0191	0.0558*	
H6	0.9882	0.5239	0.7207	0.0493*	
H8	0.8232	0.4683	0.5799	0.0380*	
H11	0.7029	0.5856	−0.0220	0.0467*	
H12	0.7878	0.6390	0.2748	0.0446*	
H14	0.5823	0.3841	0.1463	0.0417*	
H16	0.7060	0.2069	0.3270	0.0477*	
H17	0.8678	0.2556	0.4738	0.0495*	
H18	0.8882	0.3676	0.4513	0.0434*	
H21A	0.5205	0.4843	−0.1869	0.0710*	
H21B	0.5637	0.4595	−0.3768	0.0710*	
H21C	0.5917	0.5312	−0.2874	0.0710*	
H25A	0.5993	0.6656	0.5214	0.0676*	0.558 (15)
H25B	0.6661	0.7545	0.5711	0.0344*	0.442 (15)
H23A	0.3819	0.2857	0.0173	0.0352*	0.558 (15)
H23B	0.4538	0.3429	0.0999	0.0352*	0.558 (15)
H23C	0.4586	0.1777	0.1099	0.0430*	0.442 (15)
H23D	0.5739	0.1763	0.2283	0.0430*	0.442 (15)

### Atomic displacement parameters (Å^2^)

**Table:**

	*U*^11^	*U*^22^	*U*^33^	*U*^12^	*U*^13^	*U*^23^
O24A	0.075 (7)	0.026 (5)	0.059 (6)	0.005 (4)	0.016 (5)	0.019 (4)
O24B	0.021 (5)	0.011 (5)	0.092 (10)	0.005 (4)	0.020 (5)	0.011 (5)
O25A	0.056 (7)	0.044 (6)	0.072 (7)	−0.010 (5)	0.022 (5)	−0.019 (5)
O25B	0.023 (5)	0.027 (5)	0.036 (6)	−0.007 (4)	0.007 (4)	−0.024 (4)
O26	0.0500 (10)	0.0378 (9)	0.0382 (9)	−0.0020 (8)	0.0114 (8)	0.0018 (8)
O27A	0.063 (3)	0.031 (3)	0.051 (4)	−0.0162 (18)	0.0434 (19)	−0.0064 (16)
O27B	0.053 (4)	0.046 (4)	0.018 (5)	−0.011 (3)	0.008 (3)	−0.002 (3)
N23A	0.041 (3)	0.028 (3)	0.019 (4)	−0.010 (2)	0.008 (2)	−0.0085 (18)
N23B	0.058 (4)	0.029 (3)	0.034 (4)	−0.017 (3)	0.037 (3)	−0.004 (2)
C1	0.0366 (12)	0.0280 (11)	0.0404 (13)	−0.0046 (10)	0.0148 (10)	0.0007 (10)
C2	0.0403 (13)	0.0293 (12)	0.0439 (14)	−0.0054 (10)	0.0181 (11)	0.0012 (10)
C3	0.0524 (16)	0.0336 (13)	0.0480 (15)	−0.0097 (11)	0.0199 (13)	−0.0023 (11)
C4	0.0489 (15)	0.0483 (15)	0.0432 (14)	−0.0182 (13)	0.0110 (12)	−0.0042 (12)
C5	0.0377 (14)	0.0498 (15)	0.0501 (15)	−0.0048 (12)	0.0074 (12)	0.0027 (13)
C6	0.0383 (13)	0.0379 (13)	0.0490 (15)	0.0003 (11)	0.0144 (11)	0.0010 (11)
C7	0.0329 (12)	0.0282 (11)	0.0403 (13)	0.0014 (9)	0.0146 (10)	0.0002 (10)
C8	0.0353 (12)	0.0265 (11)	0.0342 (12)	0.0051 (9)	0.0104 (10)	0.0069 (9)
C9	0.0330 (12)	0.0257 (11)	0.0351 (12)	0.0027 (9)	0.0100 (10)	0.0030 (9)
C10	0.0324 (12)	0.0301 (11)	0.0356 (12)	0.0027 (10)	0.0075 (10)	0.0072 (10)
C11	0.0424 (14)	0.0328 (12)	0.0415 (14)	0.0054 (10)	0.0098 (11)	0.0139 (11)
C12	0.0414 (13)	0.0238 (11)	0.0482 (14)	0.0016 (10)	0.0145 (11)	0.0062 (10)
C13	0.0449 (14)	0.0267 (11)	0.0272 (11)	0.0030 (10)	0.0103 (10)	0.0038 (9)
C14	0.0467 (14)	0.0303 (11)	0.0287 (12)	0.0013 (10)	0.0121 (10)	0.0041 (9)
C15	0.0552 (15)	0.0303 (12)	0.0303 (12)	−0.0053 (11)	0.0193 (11)	−0.0022 (10)
C16	0.0633 (16)	0.0244 (11)	0.0379 (13)	0.0015 (11)	0.0243 (12)	0.0041 (10)
C17	0.0580 (16)	0.0304 (12)	0.0379 (13)	0.0096 (11)	0.0167 (12)	0.0056 (10)
C18	0.0452 (14)	0.0331 (12)	0.0300 (12)	0.0032 (10)	0.0086 (10)	0.0027 (10)
C19A	0.0482 (15)	0.0301 (12)	0.0418 (14)	0.0008 (11)	0.0212 (12)	0.0014 (11)
C19B	0.0482 (15)	0.0301 (12)	0.0418 (14)	0.0008 (11)	0.0212 (12)	0.0014 (11)
C20	0.0422 (13)	0.0323 (12)	0.0367 (13)	−0.0045 (10)	0.0073 (11)	0.0100 (10)
C21	0.0612 (18)	0.0556 (17)	0.0493 (16)	0.0034 (14)	−0.0085 (14)	0.0086 (14)
C22A	0.0607 (16)	0.0354 (13)	0.0330 (13)	−0.0072 (12)	0.0267 (12)	−0.0012 (11)
C22B	0.0607 (16)	0.0354 (13)	0.0330 (13)	−0.0072 (12)	0.0267 (12)	−0.0012 (11)

### Geometric parameters (Å, º)

**Table:**

O24A—C19A	1.242 (8)	C14—C15	1.395 (3)
O24B—C19B	1.243 (8)	C15—C16	1.385 (4)
O25A—C19A	1.296 (9)	C15—C22A	1.478 (3)
O25B—C19B	1.295 (8)	C15—C22B	1.478 (3)
O26—C20	1.217 (3)	C16—C17	1.382 (4)
O27A—C22A	1.250 (3)	C17—C18	1.386 (3)
O27B—C22B	1.250 (6)	C20—C21	1.498 (4)
N23A—C22A	1.320 (3)	O25A—H25A	0.840
N23B—C22B	1.320 (3)	O25B—H25B	0.840
C1—C2	1.407 (4)	N23A—H23A	0.880
C1—C6	1.399 (3)	N23A—H23B	0.880
C1—C7	1.486 (3)	N23B—H23C	0.880
C2—C3	1.390 (3)	N23B—H23D	0.880
C2—C19A	1.487 (3)	C3—H3	0.950
C2—C19B	1.487 (3)	C4—H4	0.950
C3—C4	1.379 (4)	C5—H5	0.950
C4—C5	1.376 (4)	C6—H6	0.950
C5—C6	1.381 (4)	C8—H8	0.950
C7—C8	1.393 (3)	C11—H11	0.950
C7—C12	1.393 (3)	C12—H12	0.950
C8—C9	1.389 (3)	C14—H14	0.950
C9—C10	1.409 (3)	C16—H16	0.950
C9—C13	1.490 (3)	C17—H17	0.950
C10—C11	1.398 (3)	C18—H18	0.950
C10—C20	1.494 (3)	C21—H21A	0.980
C11—C12	1.376 (3)	C21—H21B	0.980
C13—C14	1.380 (3)	C21—H21C	0.980
C13—C18	1.396 (3)		
			
O24A···C1	3.574 (8)	N23B···H14^ix^	3.5997
O24A···C3	2.824 (8)	N23B···H21B^ix^	3.3147
O24B···C1	2.924 (8)	N23B···H25B^viii^	2.7850
O24B···C3	3.468 (10)	N23B···H25B^iv^	3.4966
O24B···C7	2.939 (11)	C1···H11^x^	3.4469
O24B···C12	3.016 (15)	C1···H18^vi^	3.5052
O25A···C1	2.943 (9)	C2···H3^i^	3.4055
O25A···C3	3.537 (10)	C2···H11^x^	3.0228
O25A···C7	2.913 (11)	C3···H11^x^	3.3268
O25A···C12	2.960 (14)	C3···H12^x^	3.2816
O25B···C3	2.854 (8)	C3···H17^xiii^	3.3647
O26···C9	2.979 (3)	C4···H12^x^	3.5193
O26···C11	3.465 (3)	C4···H17^xiii^	2.8615
O26···C13	3.003 (3)	C4···H18^xiv^	3.5814
O26···C14	3.131 (3)	C5···H6^xiv^	3.3034
O27A···C16	2.831 (5)	C5···H8^xiv^	3.5616
O27B···C14	2.814 (5)	C5···H18^vi^	3.2642
N23A···C14	2.765 (4)	C6···H5^xiv^	3.2642
N23B···C16	2.756 (4)	C6···H18^vi^	3.0479
C1···C4	2.807 (4)	C7···H6^vi^	3.5474
C2···C5	2.775 (4)	C8···H6^vi^	3.2895
C2···C12	3.153 (4)	C8···H21B^x^	3.5940
C3···C6	2.749 (4)	C9···H5^vi^	3.3115
C6···C8	2.993 (3)	C9···H6^vi^	3.4150
C7···C10	2.817 (3)	C10···H5^vi^	3.1732
C7···C19A	3.042 (3)	C10···H21A^xii^	3.3333
C7···C19B	3.042 (3)	C11···H21A^xii^	3.5766
C8···C11	2.746 (4)	C11···H23A^xii^	3.4767
C8···C18	3.017 (3)	C11···H23B^xii^	3.1379
C9···C12	2.795 (3)	C12···H25B^i^	3.5920
C10···C14	3.122 (3)	C12···H23A^xii^	3.5335
C11···C21	3.090 (4)	C13···H5^vi^	3.5754
C12···C19A	3.148 (4)	C14···H16^v^	3.1983
C12···C19B	3.148 (4)	C14···H23D^v^	3.2254
C13···C16	2.793 (3)	C15···H16^v^	3.0667
C13···C20	3.006 (4)	C15···H23D^v^	3.2963
C14···C17	2.766 (4)	C16···H16^v^	3.3930
C14···C20	3.114 (4)	C16···H16^ix^	3.5377
C15···C18	2.764 (4)	C16···H17^v^	3.4310
O24A···O24A^i^	3.385 (14)	C17···H4^xv^	3.1856
O24A···O24A^ii^	3.385 (14)	C17···H4^xiv^	3.4373
O24A···O27A^iii^	3.322 (8)	C17···H16^ix^	3.4376
O24A···N23A^iii^	2.661 (8)	C17···H17^v^	3.2638
O24A···N23A^iv^	3.468 (12)	C18···H4^xiv^	3.3127
O24A···C3^i^	3.415 (10)	C19A···H3^i^	3.2358
O24A···C12^ii^	3.576 (8)	C19A···H11^x^	3.1387
O24A···C22A^iii^	3.385 (8)	C19A···H23A^iii^	2.6965
O24B···O27B^iii^	3.398 (8)	C19A···H23A^iv^	3.1779
O24B···O27B^iv^	3.535 (19)	C19A···H23B^iv^	3.4180
O24B···N23B^iii^	2.677 (10)	C19B···H3^i^	3.2358
O24B···C22B^iii^	3.427 (8)	C19B···H11^x^	3.1387
O25A···O27A^iii^	2.544 (9)	C19B···H25B^ii^	3.5495
O25A···N23A^iii^	3.537 (8)	C19B···H23C^iii^	2.6448
O25A···N23A^iv^	3.255 (17)	C20···H5^vi^	3.0855
O25A···C22A^iii^	3.404 (8)	C20···H8^vii^	3.1757
O25B···O25B^i^	3.374 (14)	C20···H16^v^	3.5037
O25B···O25B^ii^	3.374 (14)	C20···H23D^v^	3.1993
O25B···O27B^iii^	2.546 (8)	C21···H14^xii^	3.5891
O25B···N23B^iii^	3.459 (8)	C21···H21A^xii^	3.5627
O25B···C3^i^	3.404 (9)	C21···H21B^xi^	2.9537
O25B···C12^ii^	3.527 (8)	C21···H21C^xi^	3.3709
O25B···C22B^iii^	3.369 (7)	C21···H23D^v^	3.3449
O26···N23B^v^	3.537 (4)	C22A···H16^v^	3.5231
O26···C5^vi^	3.265 (3)	C22A···H25A^viii^	2.5981
O26···C8^vii^	3.421 (3)	C22A···H25A^iv^	3.5196
O26···C16^v^	3.439 (3)	C22B···H16^v^	3.5231
O27A···O24A^viii^	3.322 (8)	C22B···H25B^viii^	2.5455
O27A···O25A^viii^	2.544 (9)	C22B···H23D^v^	3.1807
O27A···N23A^v^	3.410 (12)	H3···O24A^ii^	2.6611
O27A···N23A^ix^	3.568 (12)	H3···O25B^ii^	2.6611
O27A···C14^v^	3.592 (8)	H3···C2^ii^	3.4055
O27A···C15^v^	3.209 (9)	H3···C19A^ii^	3.2358
O27A···C19A^viii^	3.314 (4)	H3···C19B^ii^	3.2358
O27A···C22A^v^	3.282 (9)	H3···H11^x^	3.5974
O27B···O24B^viii^	3.398 (8)	H3···H12^x^	3.1803
O27B···O24B^iv^	3.535 (19)	H3···H12^ii^	3.2642
O27B···O25B^viii^	2.546 (8)	H3···H17^xiii^	3.3367
O27B···N23B^v^	2.860 (14)	H3···H25B^ii^	3.1162
O27B···C19B^viii^	3.354 (5)	H3···H23A^iv^	3.4509
O27B···C22B^v^	3.305 (11)	H4···C17^xiii^	3.1856
N23A···O24A^viii^	2.661 (8)	H4···C17^xiv^	3.4373
N23A···O24A^iv^	3.468 (12)	H4···C18^xiv^	3.3127
N23A···O25A^viii^	3.537 (8)	H4···H12^x^	3.5653
N23A···O25A^iv^	3.255 (17)	H4···H16^xiii^	3.4913
N23A···O27A^v^	3.568 (12)	H4···H17^xiii^	2.4525
N23A···O27A^ix^	3.410 (12)	H4···H17^xiv^	3.0649
N23A···C19A^viii^	3.465 (4)	H4···H18^xiv^	2.8227
N23A···C19A^iv^	3.424 (8)	H5···O26^vi^	2.4442
N23B···O24B^viii^	2.677 (10)	H5···C6^xiv^	3.2642
N23B···O25B^viii^	3.459 (8)	H5···C9^vi^	3.3115
N23B···O26^ix^	3.537 (4)	H5···C10^vi^	3.1732
N23B···O27B^ix^	2.860 (14)	H5···C13^vi^	3.5754
N23B···C15^v^	3.577 (9)	H5···C20^vi^	3.0855
N23B···C19B^viii^	3.424 (5)	H5···H6^xiv^	2.9459
N23B···C22B^ix^	3.247 (9)	H5···H8^xiv^	2.7928
C3···O24A^ii^	3.415 (10)	H5···H18^vi^	3.4956
C3···O25B^ii^	3.404 (9)	H6···C5^xiv^	3.3034
C5···O26^vi^	3.265 (3)	H6···C7^vi^	3.5474
C8···O26^x^	3.421 (3)	H6···C8^vi^	3.2895
C12···O24A^i^	3.576 (8)	H6···C9^vi^	3.4150
C12···O25B^i^	3.527 (8)	H6···H5^xiv^	2.9459
C14···O27A^ix^	3.592 (8)	H6···H6^vi^	3.2114
C15···O27A^ix^	3.209 (9)	H6···H8^vi^	3.5529
C15···N23B^ix^	3.577 (9)	H6···H18^vi^	3.1384
C15···C16^v^	3.294 (4)	H8···O26^x^	2.6948
C15···C22A^ix^	3.492 (4)	H8···C5^xiv^	3.5616
C15···C22B^ix^	3.492 (4)	H8···C20^x^	3.1757
C16···O26^ix^	3.439 (3)	H8···H5^xiv^	2.7928
C16···C15^ix^	3.294 (4)	H8···H6^vi^	3.5529
C16···C16^v^	3.364 (4)	H8···H21B^x^	3.4797
C16···C16^ix^	3.364 (4)	H8···H21C^x^	3.5978
C16···C17^v^	3.397 (4)	H11···O24B^vii^	2.9092
C16···C22A^ix^	3.594 (4)	H11···O25A^vii^	2.9801
C16···C22B^ix^	3.594 (4)	H11···O27B^xii^	3.1717
C17···C16^ix^	3.397 (4)	H11···N23A^xii^	2.9961
C19A···O27A^iii^	3.314 (4)	H11···C1^vii^	3.4469
C19A···N23A^iii^	3.465 (4)	H11···C2^vii^	3.0228
C19A···N23A^iv^	3.424 (8)	H11···C3^vii^	3.3268
C19B···O27B^iii^	3.354 (5)	H11···C19A^vii^	3.1387
C19B···N23B^iii^	3.424 (5)	H11···C19B^vii^	3.1387
C21···C21^xi^	3.524 (4)	H11···H3^vii^	3.5974
C22A···O24A^viii^	3.385 (8)	H11···H25A^vii^	3.4568
C22A···O25A^viii^	3.404 (8)	H11···H25B^i^	3.4075
C22A···O27A^ix^	3.282 (9)	H11···H23A^xii^	2.8749
C22A···C15^v^	3.492 (4)	H11···H23B^xii^	2.4740
C22A···C16^v^	3.594 (4)	H12···O24A^i^	2.7077
C22A···C22A^v^	3.393 (4)	H12···O25B^i^	2.6608
C22A···C22A^ix^	3.393 (4)	H12···O27B^xii^	3.5614
C22B···O24B^viii^	3.427 (8)	H12···N23A^xii^	3.5845
C22B···O25B^viii^	3.369 (7)	H12···C3^vii^	3.2816
C22B···O27B^ix^	3.305 (11)	H12···C4^vii^	3.5193
C22B···N23B^v^	3.247 (9)	H12···H3^vii^	3.1803
C22B···C15^v^	3.492 (4)	H12···H3^i^	3.2642
C22B···C16^v^	3.594 (4)	H12···H4^vii^	3.5653
C22B···C22B^v^	3.393 (4)	H12···H25B^i^	2.8558
C22B···C22B^ix^	3.393 (4)	H12···H23A^xii^	3.0095
O24A···H3	2.5591	H12···H23B^xii^	3.5480
O24A···H12	3.3956	H12···H23C^iii^	3.5825
O24A···H25A	2.2753	H14···N23B^v^	3.5997
O24B···H12	2.9356	H14···C21^xii^	3.5891
O24B···H25B	2.2756	H14···H16^v^	3.5243
O25A···H12	2.8862	H14···H21A^xii^	3.0664
O25B···H3	2.5728	H14···H21C^xii^	3.1902
O25B···H12	3.4386	H14···H23D^v^	3.0506
O26···H14	3.1162	H16···O26^ix^	2.5039
O26···H21A	2.9916	H16···C14^ix^	3.1983
O26···H21B	2.4516	H16···C15^ix^	3.0667
O26···H21C	2.9767	H16···C16^v^	3.5377
O27A···H16	2.5604	H16···C16^ix^	3.3930
O27A···H23A	2.4581	H16···C17^v^	3.4376
O27A···H23B	3.0392	H16···C20^ix^	3.5037
O27B···H14	2.5151	H16···C22A^ix^	3.5231
O27B···H23C	2.4575	H16···C22B^ix^	3.5231
O27B···H23D	3.0390	H16···H4^xv^	3.4913
N23A···H14	2.4416	H16···H14^ix^	3.5243
N23B···H16	2.4411	H17···C3^xv^	3.3647
C1···H3	3.2766	H17···C4^xv^	2.8615
C1···H5	3.2792	H17···C16^ix^	3.4310
C1···H8	2.6189	H17···C17^ix^	3.2638
C1···H12	2.7007	H17···H3^xv^	3.3367
C2···H4	3.2692	H17···H4^xv^	2.4525
C2···H6	3.2603	H17···H4^xiv^	3.0649
C2···H12	2.9560	H17···H17^v^	3.3703
C2···H25A	3.0999	H17···H17^ix^	3.3703
C2···H25B	3.1025	H18···C1^vi^	3.5052
C3···H5	3.2381	H18···C4^xiv^	3.5814
C4···H6	3.2418	H18···C5^vi^	3.2642
C5···H3	3.2344	H18···C6^vi^	3.0479
C6···H4	3.2495	H18···H4^xiv^	2.8227
C6···H8	2.7695	H18···H5^vi^	3.4956
C7···H6	2.6147	H18···H6^vi^	3.1384
C7···H11	3.2568	H21A···C10^xii^	3.3333
C8···H6	2.7458	H21A···C11^xii^	3.5766
C8···H12	3.2501	H21A···C21^xii^	3.5627
C8···H18	2.8136	H21A···H14^xii^	3.0664
C9···H11	3.2713	H21A···H21A^xii^	2.7717
C9···H14	2.6638	H21A···H21B^xi^	3.0955
C9···H18	2.6478	H21A···H21C^xi^	3.4959
C10···H8	3.2622	H21A···H23D^v^	3.4547
C10···H12	3.2741	H21B···O24B^xii^	3.5677
C10···H14	2.9289	H21B···O25A^xii^	3.5143
C10···H21A	2.7967	H21B···O27A^v^	3.3553
C10···H21B	3.3846	H21B···N23B^v^	3.3147
C10···H21C	2.7960	H21B···C8^vii^	3.5940
C11···H21A	3.3309	H21B···C21^xi^	2.9537
C11···H21C	2.8476	H21B···H8^vii^	3.4797
C12···H8	3.2438	H21B···H21A^xi^	3.0955
C12···H25A	3.5025	H21B···H21B^xi^	2.6366
C13···H8	2.6019	H21B···H21C^xi^	2.6513
C13···H17	3.2704	H21B···H25A^xii^	3.3333
C14···H16	3.2687	H21B···H23C^v^	3.1327
C14···H18	3.2477	H21B···H23D^v^	2.8792
C14···H23B	2.3993	H21C···O24B^vii^	2.7212
C15···H17	3.2482	H21C···O25A^vii^	2.7275
C15···H23A	3.1925	H21C···C21^xi^	3.3709
C15···H23B	2.4815	H21C···H8^vii^	3.5978
C15···H23C	3.1858	H21C···H14^xii^	3.1902
C15···H23D	2.4688	H21C···H21A^xi^	3.4959
C16···H14	3.2640	H21C···H21B^xi^	2.6513
C16···H18	3.2552	H21C···H21C^xi^	3.4970
C16···H23D	2.3755	H21C···H25A^vii^	3.0628
C18···H8	2.7759	H21C···H23B^xii^	3.0047
C18···H14	3.2497	H25A···O27A^iii^	1.7163
C18···H16	3.2589	H25A···N23A^iii^	2.8889
C19A···H3	2.5904	H25A···N23A^iv^	2.9827
C19A···H12	2.7932	H25A···C22A^iii^	2.5981
C19B···H3	2.5904	H25A···C22A^iv^	3.5196
C19B···H12	2.7932	H25A···H11^x^	3.4568
C20···H11	2.6426	H25A···H21B^xii^	3.3333
C20···H14	2.8136	H25A···H21C^x^	3.0628
C21···H11	2.8220	H25A···H23A^iii^	2.5047
C21···H14	3.3967	H25A···H23A^iv^	3.2114
C22A···H14	2.6320	H25A···H23B^iv^	2.8039
C22A···H16	2.6522	H25B···O25B^i^	3.2598
C22B···H14	2.6320	H25B···O27B^iii^	1.7200
C22B···H16	2.6522	H25B···O27B^iv^	3.5315
H3···H4	2.3252	H25B···N23B^iii^	2.7850
H3···H25B	3.3443	H25B···N23B^iv^	3.4966
H4···H5	2.3286	H25B···C12^ii^	3.5920
H5···H6	2.3172	H25B···C19B^i^	3.5495
H6···H8	2.4218	H25B···C22B^iii^	2.5455
H8···H18	2.4745	H25B···H3^i^	3.1162
H11···H12	2.3115	H25B···H11^ii^	3.4075
H11···H21A	3.1583	H25B···H12^ii^	2.8558
H11···H21C	2.2998	H25B···H25B^i^	3.3673
H12···H25A	3.3341	H25B···H25B^ii^	3.3673
H14···H21A	3.0075	H25B···H23C^iii^	2.3807
H14···H23A	3.2571	H25B···H23C^iv^	3.3038
H14···H23B	1.8390	H23A···O24A^viii^	1.7981
H16···H17	2.3329	H23A···O24A^iv^	3.1195
H16···H23C	3.2639	H23A···O25A^viii^	3.0009
H16···H23D	1.8052	H23A···O25A^iv^	3.3417
H17···H18	2.3289	H23A···C11^xii^	3.4767
O24A···H3^i^	2.6611	H23A···C12^xii^	3.5335
O24A···H12^ii^	2.7077	H23A···C19A^viii^	2.6965
O24A···H23A^iii^	1.7981	H23A···C19A^iv^	3.1779
O24A···H23A^iv^	3.1195	H23A···H3^iv^	3.4509
O24A···H23B^iii^	3.2737	H23A···H11^xii^	2.8749
O24B···H11^x^	2.9092	H23A···H12^xii^	3.0095
O24B···H21B^xii^	3.5677	H23A···H25A^viii^	2.5047
O24B···H21C^x^	2.7212	H23A···H25A^iv^	3.2114
O24B···H23C^iii^	1.8297	H23B···O24A^viii^	3.2737
O24B···H23D^iii^	3.2621	H23B···O25A^iv^	2.9887
O25A···H11^x^	2.9801	H23B···O27A^ix^	3.3768
O25A···H21B^xii^	3.5143	H23B···C11^xii^	3.1379
O25A···H21C^x^	2.7275	H23B···C19A^iv^	3.4180
O25A···H23A^iii^	3.0009	H23B···H11^xii^	2.4740
O25A···H23A^iv^	3.3417	H23B···H12^xii^	3.5480
O25A···H23B^iv^	2.9887	H23B···H21C^xii^	3.0047
O25B···H3^i^	2.6611	H23B···H25A^iv^	2.8039
O25B···H12^ii^	2.6608	H23C···O24B^viii^	1.8297
O25B···H25B^ii^	3.2598	H23C···O25B^viii^	2.9043
O25B···H23C^iii^	2.9043	H23C···O27B^ix^	2.9182
O26···H5^vi^	2.4442	H23C···C19B^viii^	2.6448
O26···H8^vii^	2.6948	H23C···H12^viii^	3.5825
O26···H16^v^	2.5039	H23C···H21B^ix^	3.1327
O26···H23D^v^	2.6860	H23C···H25B^viii^	2.3807
O27A···H21B^ix^	3.3553	H23C···H25B^iv^	3.3038
O27A···H25A^viii^	1.7163	H23D···O24B^viii^	3.2621
O27A···H23B^v^	3.3768	H23D···O26^ix^	2.6860
O27B···H11^xii^	3.1717	H23D···O27B^ix^	2.9533
O27B···H12^xii^	3.5614	H23D···C14^ix^	3.2254
O27B···H25B^viii^	1.7200	H23D···C15^ix^	3.2963
O27B···H25B^iv^	3.5315	H23D···C20^ix^	3.1993
O27B···H23C^v^	2.9182	H23D···C21^ix^	3.3449
O27B···H23D^v^	2.9533	H23D···C22B^ix^	3.1807
N23A···H11^xii^	2.9961	H23D···H14^ix^	3.0506
N23A···H12^xii^	3.5845	H23D···H21A^ix^	3.4547
N23A···H25A^viii^	2.8889	H23D···H21B^ix^	2.8792
N23A···H25A^iv^	2.9827		
			
C2—C1—C6	117.59 (19)	C10—C20—C21	118.6 (2)
C2—C1—C7	123.86 (19)	O27A—C22A—N23A	123.0 (3)
C6—C1—C7	118.5 (2)	O27A—C22A—C15	120.5 (2)
C1—C2—C3	119.9 (2)	N23A—C22A—C15	116.41 (18)
C1—C2—C19A	122.55 (18)	O27B—C22B—N23B	123.0 (3)
C1—C2—C19B	122.55 (18)	O27B—C22B—C15	121.2 (3)
C3—C2—C19A	117.3 (2)	N23B—C22B—C15	115.8 (3)
C3—C2—C19B	117.3 (2)	C19A—O25A—H25A	109.468
C2—C3—C4	121.2 (3)	C19B—O25B—H25B	109.472
C3—C4—C5	119.4 (3)	C22A—N23A—H23A	119.991
C4—C5—C6	120.2 (3)	C22A—N23A—H23B	119.999
C1—C6—C5	121.6 (3)	H23A—N23A—H23B	120.010
C1—C7—C8	119.34 (19)	C22B—N23B—H23C	120.003
C1—C7—C12	122.66 (19)	C22B—N23B—H23D	119.996
C8—C7—C12	117.97 (19)	H23C—N23B—H23D	120.001
C7—C8—C9	122.6 (2)	C2—C3—H3	119.404
C8—C9—C10	118.71 (19)	C4—C3—H3	119.397
C8—C9—C13	118.60 (19)	C3—C4—H4	120.310
C10—C9—C13	122.65 (18)	C5—C4—H4	120.297
C9—C10—C11	118.59 (19)	C4—C5—H5	119.883
C9—C10—C20	121.66 (19)	C6—C5—H5	119.871
C11—C10—C20	119.4 (2)	C1—C6—H6	119.186
C10—C11—C12	121.6 (2)	C5—C6—H6	119.188
C7—C12—C11	120.5 (2)	C7—C8—H8	118.701
C9—C13—C14	121.38 (19)	C9—C8—H8	118.708
C9—C13—C18	119.75 (19)	C10—C11—H11	119.201
C14—C13—C18	118.8 (2)	C12—C11—H11	119.198
C13—C14—C15	120.6 (2)	C7—C12—H12	119.750
C14—C15—C16	120.1 (2)	C11—C12—H12	119.749
C14—C15—C22A	119.38 (19)	C13—C14—H14	119.694
C14—C15—C22B	119.38 (19)	C15—C14—H14	119.708
C16—C15—C22A	120.48 (18)	C15—C16—H16	120.253
C16—C15—C22B	120.48 (18)	C17—C16—H16	120.260
C15—C16—C17	119.5 (2)	C16—C17—H17	119.827
C16—C17—C18	120.4 (2)	C18—C17—H17	119.822
C13—C18—C17	120.5 (2)	C13—C18—H18	119.731
O24A—C19A—O25A	123.5 (5)	C17—C18—H18	119.720
O24A—C19A—C2	118.1 (4)	C20—C21—H21A	109.468
O25A—C19A—C2	118.4 (4)	C20—C21—H21B	109.469
O24B—C19B—O25B	123.4 (5)	C20—C21—H21C	109.470
O24B—C19B—C2	117.8 (4)	H21A—C21—H21B	109.473
O25B—C19B—C2	118.5 (4)	H21A—C21—H21C	109.473
O26—C20—C10	120.51 (19)	H21B—C21—H21C	109.475
O26—C20—C21	120.8 (2)		
			
H25A—O25A—C19A—O24A	−5.5	H8—C8—C9—C13	1.5
H25A—O25A—C19A—C2	173.8	C8—C9—C10—C11	−1.0 (3)
H25B—O25B—C19B—O24B	−6.2	C8—C9—C10—C20	172.06 (19)
H25B—O25B—C19B—C2	179.2	C8—C9—C13—C14	129.7 (2)
H23A—N23A—C22A—O27A	0.0	C8—C9—C13—C18	−48.4 (3)
H23A—N23A—C22A—C15	179.0	C10—C9—C13—C14	−48.3 (4)
H23B—N23A—C22A—O27A	−180.0	C10—C9—C13—C18	133.6 (2)
H23B—N23A—C22A—C15	−1.0	C13—C9—C10—C11	176.98 (19)
H23C—N23B—C22B—O27B	−0.0	C13—C9—C10—C20	−10.0 (4)
H23C—N23B—C22B—C15	178.0	C9—C10—C11—C12	2.2 (4)
H23D—N23B—C22B—O27B	180.0	C9—C10—C11—H11	−177.8
H23D—N23B—C22B—C15	−2.0	C9—C10—C20—O26	−44.3 (4)
C2—C1—C6—C5	0.3 (4)	C9—C10—C20—C21	137.2 (2)
C2—C1—C6—H6	−179.7	C11—C10—C20—O26	128.6 (3)
C6—C1—C2—C3	1.1 (4)	C11—C10—C20—C21	−49.8 (3)
C6—C1—C2—C19A	−173.7 (2)	C20—C10—C11—C12	−170.9 (2)
C6—C1—C2—C19B	−173.7 (2)	C20—C10—C11—H11	9.0
C2—C1—C7—C8	−137.0 (3)	C10—C11—C12—C7	−2.1 (4)
C2—C1—C7—C12	45.2 (4)	C10—C11—C12—H12	177.9
C7—C1—C2—C3	−175.7 (2)	H11—C11—C12—C7	177.9
C7—C1—C2—C19A	9.4 (4)	H11—C11—C12—H12	−2.0
C7—C1—C2—C19B	9.4 (4)	C9—C13—C14—C15	179.61 (19)
C6—C1—C7—C8	46.2 (4)	C9—C13—C14—H14	−0.4
C6—C1—C7—C12	−131.6 (3)	C9—C13—C18—C17	179.93 (19)
C7—C1—C6—C5	177.3 (2)	C9—C13—C18—H18	−0.1
C7—C1—C6—H6	−2.7	C14—C13—C18—C17	1.8 (4)
C1—C2—C3—C4	−2.2 (4)	C14—C13—C18—H18	−178.2
C1—C2—C3—H3	177.8	C18—C13—C14—C15	−2.3 (4)
C1—C2—C19A—O24A	−146.0 (3)	C18—C13—C14—H14	177.7
C1—C2—C19A—O25A	34.7 (4)	C13—C14—C15—C16	1.1 (4)
C1—C2—C19B—O24B	38.5 (4)	C13—C14—C15—C22A	−177.7 (2)
C1—C2—C19B—O25B	−146.6 (3)	C13—C14—C15—C22B	−177.7 (2)
C3—C2—C19A—O24A	39.0 (4)	H14—C14—C15—C16	−178.9
C3—C2—C19A—O25A	−140.3 (3)	H14—C14—C15—C22A	2.3
C19A—C2—C3—C4	172.9 (2)	H14—C14—C15—C22B	2.3
C19A—C2—C3—H3	−7.1	C14—C15—C16—C17	0.6 (4)
C3—C2—C19B—O24B	−136.5 (3)	C14—C15—C16—H16	−179.4
C3—C2—C19B—O25B	38.4 (4)	C14—C15—C22A—O27A	164.9 (2)
C19B—C2—C3—C4	172.9 (2)	C14—C15—C22A—N23A	−14.1 (4)
C19B—C2—C3—H3	−7.1	C14—C15—C22B—O27B	2.7 (4)
C2—C3—C4—C5	1.8 (4)	C14—C15—C22B—N23B	−175.4 (2)
C2—C3—C4—H4	−178.2	C16—C15—C22A—O27A	−14.0 (4)
H3—C3—C4—C5	−178.2	C16—C15—C22A—N23A	167.0 (2)
H3—C3—C4—H4	1.8	C22A—C15—C16—C17	179.4 (2)
C3—C4—C5—C6	−0.4 (4)	C22A—C15—C16—H16	−0.6
C3—C4—C5—H5	179.6	C16—C15—C22B—O27B	−176.1 (3)
H4—C4—C5—C6	179.6	C16—C15—C22B—N23B	5.8 (4)
H4—C4—C5—H5	−0.4	C22B—C15—C16—C17	179.4 (2)
C4—C5—C6—C1	−0.7 (4)	C22B—C15—C16—H16	−0.6
C4—C5—C6—H6	179.3	C15—C16—C17—C18	−1.1 (4)
H5—C5—C6—C1	179.3	C15—C16—C17—H17	178.9
H5—C5—C6—H6	−0.7	H16—C16—C17—C18	178.9
C1—C7—C8—C9	−177.22 (19)	H16—C16—C17—H17	−1.1
C1—C7—C8—H8	2.8	C16—C17—C18—C13	−0.1 (4)
C1—C7—C12—C11	178.4 (2)	C16—C17—C18—H18	179.9
C1—C7—C12—H12	−1.6	H17—C17—C18—C13	179.9
C8—C7—C12—C11	0.6 (4)	H17—C17—C18—H18	−0.1
C8—C7—C12—H12	−179.4	O26—C20—C21—H21A	121.5
C12—C7—C8—C9	0.7 (4)	O26—C20—C21—H21B	1.5
C12—C7—C8—H8	−179.3	O26—C20—C21—H21C	−118.5
C7—C8—C9—C10	−0.5 (4)	C10—C20—C21—H21A	−60.1
C7—C8—C9—C13	−178.49 (19)	C10—C20—C21—H21B	179.9
H8—C8—C9—C10	179.5	C10—C20—C21—H21C	59.9

Symmetry codes: (i) *x*, −*y*+3/2, *z*−1/2; (ii) *x*, −*y*+3/2, *z*+1/2; (iii) −*x*+1, *y*+1/2, −*z*+1/2; (iv) −*x*+1, −*y*+1, −*z*+1; (v) *x*, −*y*+1/2, *z*−1/2; (vi) −*x*+2, −*y*+1, −*z*+1; (vii) *x*, *y*, *z*−1; (viii) −*x*+1, *y*−1/2, −*z*+1/2; (ix) *x*, −*y*+1/2, *z*+1/2; (x) *x*, *y*, *z*+1; (xi) −*x*+1, −*y*+1, −*z*−1; (xii) −*x*+1, −*y*+1, −*z*; (xiii) −*x*+2, *y*+1/2, −*z*+3/2; (xiv) −*x*+2, −*y*+1, −*z*+2; (xv) −*x*+2, *y*−1/2, −*z*+3/2.

### Hydrogen-bond geometry (Å, º)

**Table:**

*D*—H···*A*	*D*—H	H···*A*	*D*···*A*	*D*—H···*A*
O25*A*—H25*A*···O27*A*^iii^	0.84	1.72	2.544 (9)	168
O25*B*—H25*B*···O27*B*^iii^	0.84	1.72	2.546 (8)	167
N23*A*—H23*A*···O24*A*^viii^	0.88	1.80	2.661 (8)	166
N23*B*—H23*C*···O24*B*^viii^	0.88	1.83	2.667 (10)	161

Symmetry codes: (iii) −*x*+1, *y*+1/2, −*z*+1/2; (viii) −*x*+1, *y*−1/2, −*z*+1/2.
